# Bt technology -the way forward

**DOI:** 10.1186/1753-6561-8-S4-O26

**Published:** 2014-10-01

**Authors:** Fernando Valicente

**Affiliations:** 1 Embrapa Maize and Sorghum, Sete Lagoas, MG, Brasil

## 

*Bacillus thuringiensis *(Bt) is a Gram-positive soil bacterium, has been isolated from a range of habitats including soil, grain dust, phylloplane and dead insects. It is characterized by its ability to produce crystalline inclusions called ''endotoxin proteins" during sporulation. The crystalline inclusions (Cry and Cyt proteins), along with the spores, have a great potential to control a number of insect pests belonging to the order Lepidoptera, Diptera, Coleoptera, Hemiptera and Hymenoptera. Besides Bt may also control nematodes. Therefore, they represent a valuable tool for Integrated Pest Management (IPM). Bt is one the most important microbial biopesticides produced in the world, accounting for 1-2% of the global insecticide market and is responsible for over 90% of all biopesticide sales. The widespread occurrence of this bacterium and interest in the use of Bt products as an alternative to chemical insecticides stimulated the isolation of native Bt strains in many parts of the world. The characterization may also be used to predict insecticidal activity, determine ecological distribution, and identify new cry genes. Besides being an important component of studying Bt resources, the characterization of Cry proteins and its genotypic composition may help understanding its insecticidal activity. Bt also produces other important toxins such as: phospholipases, proteases, quinases, α exotoxins, β exotoxins, VIP (Vegetative Insecticidal Proteins), and δ endotoxins (crystalline inclusions). Bt harbors more than 72 classes of Cry protein, the most common proteins used in transgenic plants. Table 1 below shows all Bt genes present today in transgenic plants in Brazil.

**Table 1:** Bt genes currently deployed in transgenic crops in Brazil

Crop	Bt genes
Corn	Cry2Ab,1Ab, 1A.105 (1Ab, 1Ac, 1F),Cry3Bb, 1F, VIP3Aa

Cotton	Cry2Ab,1Ab,2Ae, Cry1Ac, 1F

Soybean	Cry1Ac

Abroad proteins Cry34 and 35 have been used in transgenic crops. Despite the great number of Cry proteins described and more than 600 genes from *cry1Aa1 *to *cry72*, not many different proteins have been cloned in crops nowadays. There are many different perspectives in Bt technology in Brazil besides the use in programs of transgenic crops. The demand for the use of Bt based biopesticides has increased in many Brazilian regions. Some important factors have contributed as the introduction of the new pest *Helicoverpa armigera *that has attacked crops such as maize, cotton, soybean, millet, sorghum, beans, green peppers, tomato, okra etc. Another important factor is because of "safrinha". Due to a favorable climate, crops are planted all over the year, and the farmers plant a second crop of corn or cotton called the *safrinha*. This new scheme of crop rotation is to first plant a crop of rain-fed such as rice, soybean or maize, and then after these crops are harvested, plant a second crop of soybean, sorghum or even maize. *Safrinha *can also be defined as a farming strategy whereby the farmer takes advantage of a long tropical growing season to produce two crops in a single growing season, thereby maximizing revenue per acre. So, it is paramount to continue the research on Bt to find new and more efficient genes, and produce Bt based biopesticides feasible and cheap to the market.

